# Real-time Optical Dimensional Metrology via Diffractometry for Nanofabrication

**DOI:** 10.1038/s41598-020-61975-3

**Published:** 2020-03-25

**Authors:** Guy L. Whitworth, Achille Francone, Clivia M. Sotomayor-Torres, Nikolaos Kehagias

**Affiliations:** 1 Catalan Institute of Nanoscience and Nanotechnology (ICN2), CSIC and BIST, Campus UAB, 08193 Bellaterra, Barcelona Spain; 2 Institucio Catalana de Recerca i Estudis Avancats (ICREA), 08010 Barcelona, Spain; 3 Present Address: Institut de Ciències Fotòniques (ICFO), Mediterranean Technology Park, Avinguda Carl Friedrich Gauss, 3, 08860 Castelldefels, Barcelona Spain

**Keywords:** Nanometrology, Techniques and instrumentation, Optical techniques

## Abstract

Surface patterning technologies represent a worldwide growing industry, creating smart surfaces and micro/nanoscale device. The advent of large-area, high-speed imprinting technologies has created an ever-growing need for rapid and non-destructive dimensional metrology techniques to keep pace with the speed of production. Here we present a new real-time optical scatterometry technique, applicable at the mesoscale when optical inspection produces multiple orders of diffraction. We validate this method by inspecting multiple silicon gratings with a variety of structural parameters. These measurements are cross-referenced with FIB, SEM and scanning stylus profilometry. Finally, we measure thermally imprinted structures as a function of imprinting temperature in order to demonstrate the method suitable for in-line quality control in nanoimprint lithography.

## Introduction

Smart surfaces with nano- and micro- scale features find application in a wide variety of sectors in modern society: electronics^1^, security^2^, photonics^3^, micro-optics, micro- fluidics^4^ and biomedicine^5^. High-throughput NIL technologies such as roll-to-roll, roll-to-plate, and step-and-repeat enable high-volume production of such devices and surfaces^6–9^. For quality control purposes, inspection technologies are required to be compatible with these high-speed patterning technologies, i.e., fast and suitable for large area critical dimension (CD) measurements^10–13^. Traditional powerful CD technologies such as scanning electron microscopy (SEM) and atomic force microscopy (AFM) have serious drawbacks for such an application. They are hard to insert into a production, comparatively slow, require precise alignment and are potentially destructive. For this reason, a variety of non-imaging optical technologies are used for inspection having the advantage of being fast and non-destructive.

Simple spectroscopic techniques such as reflectivity, absorption, fluorescence or Raman scattering^14,15^ can be used to determine the amount of a material present which in turn can be related to film thickness. However, for more complex structural determination, optical scatterometry has become a widely investigated and adopted technique^16–18^. Scatterometry techniques use scattered light from a surface as a function of a variable such as angle-of-incidence or wavelength. This surface scattering response is then compared to a library of simulated data created by electromagnetic (EM) modelling techniques to fit the measured response to a computational prediction a method known as inverse problem solving. Ellipsometry is an example of a powerful scatterometry technique utilising elliptical phases which can determine thin-film optical constants and layer thicknesses down to several nanometres^19^. For nanoscale CD metrology, angle- and or wavelength- dependant reflectometry is utilised, whereby the dependence of the reflected intensity of an incident beam (0^th^ order diffraction) can be fitted to an EM library, typically computed by rigorous coupled-wave analysis (RCWA)^17,20–26^. Coherent-Fourier scatterometry is a more advanced version of this technique whereby a Fourier image can be taken of the target structure using a CCD in order to obtain the reflected angle dependence in a signal shot^16,18,27^.

**Figure 1:**
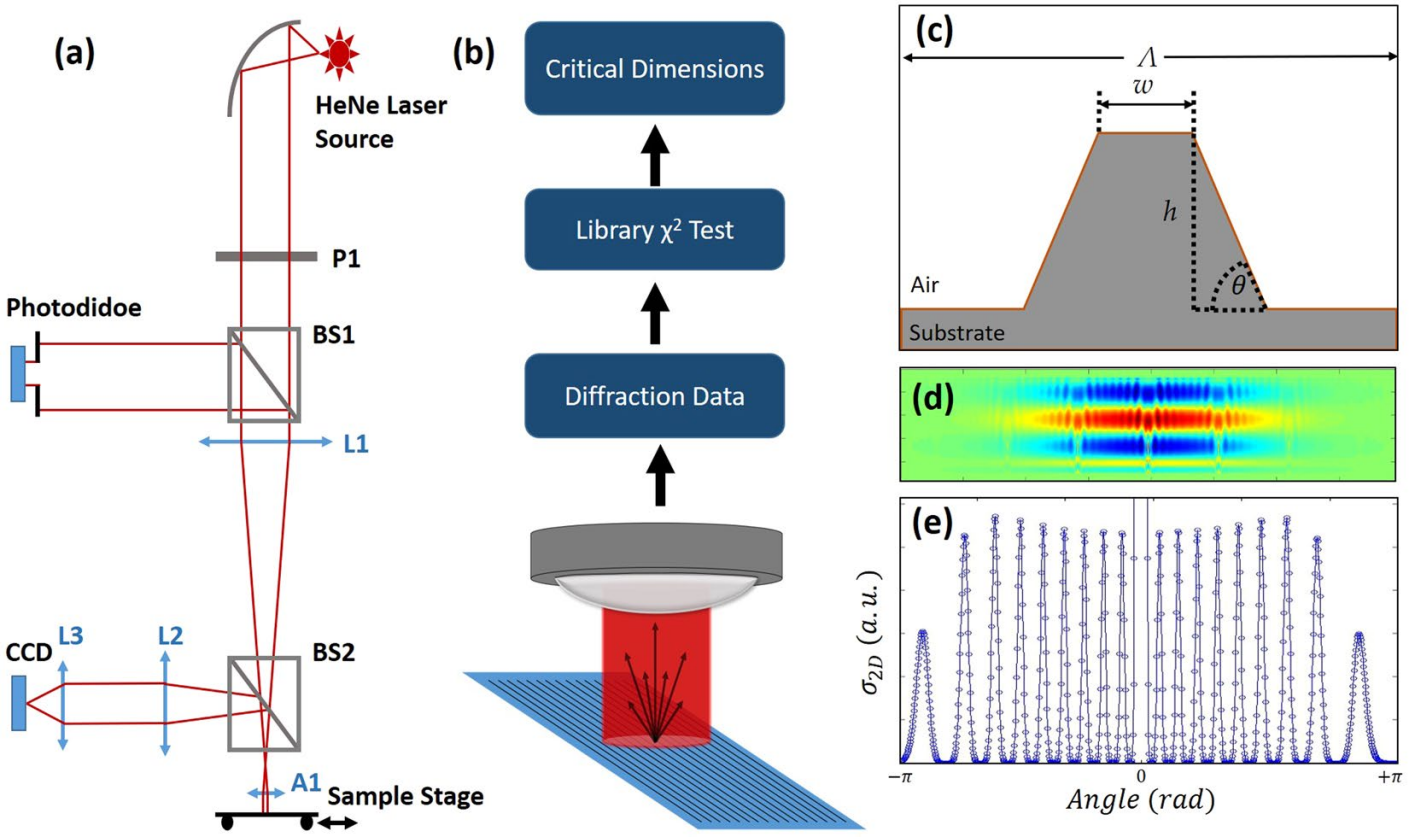
Experimental setup, metrology workflow and simulations. (**a**) An optical diagram of the diffractometry setup indicating the asphere(A1), polariser(P1) and the various lenses(L) and beamsplitters (BS). The CCD is used to collect the diffraction pattern and the photodiode for detecting the power of the 0^th^ order reflected mode. (**b**) Workflow diagram explaining the metrological process of diffractometry. (**c**) Schematic of simulated unit cell indicating the period, Λ, top-width, w, height, h, and side-wall angle θ. (**d**) Example near-field response (E_z_) of an incident Gaussian field on seven periods of a microstructure. The reflected response is then used to compute the far-field diffraction pattern (**e**) using Eq. 1.

For micro-scale NIL applications where multiple orders of diffraction exist, as is the case for diffractive optical elements or smart medical surfaces^5^, we present a scatterometry method, advanced upon our previous work^28,29^, which analyses the diffraction efficiency of all collected diffractive orders simultaneously and compares this data to a finite-difference frequency-domain (FDFD) EM library. Diffraction data in our experiment was collected with a CCD (Fig. 1(a)) and normalised to the 0th order reflection using a separate photodiode to monitor the 0^th^ order power. This data is then compared to the EM library using a χ^2^ minimisation process to compare the experimental data to the computed model to yield the top-width, *w*, height, *h*, and SWA, *θ*, measured in real-time (Fig. 1(b)). Figure 1(c) depicts a typical unit-cell used in the FDFD simulations for generating the metrology libraries. The aforementioned structural parameters for measurement are indicated along with the grating period, Λ, which remains constant in the simulations.

## Results

### Diffraction pattern simulations

Careful choice of computational parameters is required when computing the diffraction libraries as an inverse-problem measurement is only as good as the quality of the simulations. In order to predict a full spatial diffraction pattern, one must first compute the near-field response and then project this into the far-field using an equivalence principle relation (Eq. 1) resulting in a diffraction pattern^30^.

1 $${\sigma }_{2D}(\phi )\propto {|{\oint }_{L}\{{\eta }_{0}({n}_{x}^{{\prime} }{H}_{y}-{n}_{y}^{{\prime} }{H}_{x})-({n}_{y}^{{\prime} }\sin \phi +{n}_{x}^{{\prime} }\cos \phi ){E}_{z}\}{e}^{ikr{\prime} \cos (\phi -\phi {\prime} )}kdl{\prime} |}^{2}$$

This is a rectangular closed-loop line integral along the *x* and *y* edges of the simulation where the near-field response of the electric (E) and magnetic (*H*) fields are related to the 2D scattering cross-section, ${\sigma }_{2D}$ in the far-field as a function of angle, $\phi$.

Initial attempts to simulate diffraction patterns involved a single unit cell with periodic boundary-conditions. The resulting near-field was then stitched together with itself to be used for far-field projection. This method produced unrealistic diffraction patterns and was arbitrarily dependant on the number of repetitions chosen of the unit cell. The prevailing, more successful method was to simulate several unit cells (>7) and use an incident Gaussian field, surrounded by perfectly-matched layers. Whilst the simulated beam sizes (~20 μm) were not accurate to the beam waist of the experiment (~200 μm) it was observed that the simulated diffraction pattern converged after a sufficient number of unit cells (5–7) was illuminated. An example of such a near-field FDFD simulation and resultant diffraction pattern is shown in Fig. 1(d,e).

In total, five sets of samples were fabricated to test the diffractometry tool, summarised in Table 1 below. Four were fabricated by e-beam lithography and one set via thermal nanoimprinting. Each set was designed to vary different structural parameters over different ranges (see supporting information Table S1) to test the full capabilities of the metrology technique. All structures were scanned underneath the inspection beam using an automatic translation stage with a speed of 1 mm/s.

**Table 1:** Sample description and key parameters.

Sample Code	Design	Period (μm)	Area (mm x mm)	No. of Samples	Material	Key Structural Parameter
S	Step-Wise	6	40 × 5	1	Silicon	Width
T	Tapered	5	70 × 0.5	1	Silicon	Width
H	Height Variation	5	20 × 20	3	Silicon	Height
D	Defective (See SI)	5	20 × 20	4	Silicon	All
N	Nanoimprinted	5	20 × 20	9	PMMA	Height

### Step-wise structure

**Figure 2:**
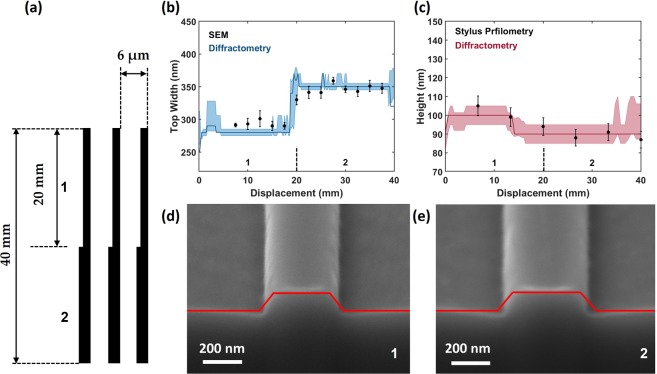
Step-wise structures. (**a**) Structural design of the 40 mm long step-wise structure with sections 1 and 2 indicated. (**b**,**c**) Diffractometry results for the top-width and height (blue and red). The black points represent the respective cross-referencing measurements of SEM and stylus profilometry. (**d**,**e**) SEM images of FIB cut cross-sections of the sections with an overlay accurate to the average diffractometry results respectively from each section.

The initial sample tested was a one dimensional, 40 mm long silicon grating with a 6 μm period, designed to have the line-width vary over a small range (~50 nm) in a step-wise manner over two different sections (shown in the schematic Fig. 2(a)). Figure 2(b,c) show the resultant top-width and height from the χ^2^ minimisation (blue and red respectively) obtained via diffractometry (SWA data can be found in supporting information Fig. S1). The coloured shaded area is the spread of possible results which are within one sigma of the χ^2^ minimum, calculated from the raw signal variation. That is to say that if there are defects or the structural dimensions are changing rapidly, the standard deviation increases and there will be multiple degenerate outcomes from the χ^2^ minimisation process within the uncertainty of the experiment.

A clear stepwise behaviour can be observed in the top-width data (blue points in Fig. 2(b)) between the two sections of the grating. Top-down SEM images were taken every 2.5 mm along the grating to measure the top linewidth (black points) as a cross-reference. This image analysis was performed by eye and the error bars are computed as the standard deviation between five separate measurements. The two sections can be seen to be stable in structural parameters and match closely to the diffractometry results. There is a small 20 nm discrepancy at 12.5 mm displacement as accompanied by a large standard deviation as measured widths via SEM indicating a defective region. The height data in Fig. 2(c), plotted in the same manner as that of the top-width, was cross-referenced using stylus profilometry. The data shows that the height of the structure varied between 90 and 100 nm along the gratings as expected from uniform etching.

To further verify the accuracy of the diffractometry, FIB cuts were made in the silicon lines. Angled SEM images (Fig. 2(d,e)) were then taken of these the separate cuts and the averaged results from the diffractometry data for each section was then overlaid onto the SEM images (red lines). The height and width data are seen to overlap well with the cross-sectional profile of the grating line, however, the SWA differs. This is assigned to the unexpected presence of over-etching at the base of the S-series gratings and a certain level of corner rounding is present in the samples. Top and bottom corner roading radii as extra simulation parameters have been omitted for simplicity in the development of the technique, as the total simulation time is increases linearly to the volume of the input n-dimensional parametric data cube.

### Tapered grating structure

**Figure 3:**
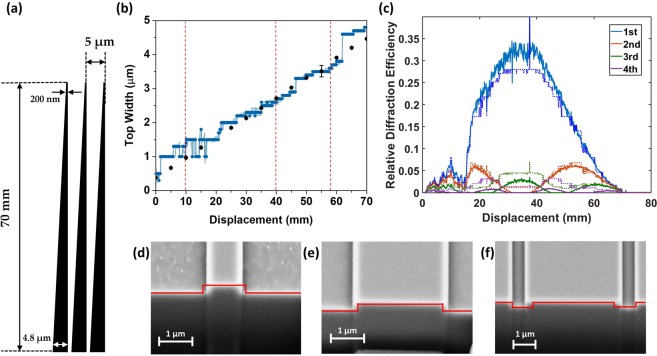
Tapered structures. (**a**) 70 mm long right-angled tapered design of gratings. (**b**) Diffractometry top-width results (blue) with SEM data (black points). Dotted red lines indicate approximately where FIB cuts were made. (**c**) Raw (solid) and fitted (dotted) diffraction data for the first 4 orders of diffraction collected as a function of the displacement along the tapers. (**d**–**f**) SEM images of FIB cut cross-sections the indicated locations with red overlays obtained from the diffractometry data.

To further test top-width sensing, a 70 mm long right-angled Tapered grating was fabricated with a period of 5 μm and designed to have the width vary from 200 nm to 4.8 μm (Fig. 3(a)). The tapered structures were passed through the optical inspection beam of the diffractometer as with the previous case to obtain real-time top-width measurement, shown in Fig. 3(b) (blue data). A clear linear trend could be observed as expected from the tapers (height and angle data can be found in supporting information Fig. S2). The measurements of the top-width were noisy in the 0–15 mm displacement region, deviating from the linear trend, also reflected in the measured relative diffraction efficiency shown Fig. 3(c) (solid lines). The noisy raw signal in this section was due to a residual polymer layer on the surface of the structure which contributed to increased scattering as seen in the optical images in supporting information Fig. S3. As before, width measurement data obtained via SEM images has been overlaid on the diffractometry data in Fig. 3(b) to verify the results (black points). Also shown in Fig. 3(c) is the resultant “fitted” diffraction efficiencies (dotted lines) as predicted by the FDFD solver.

Figure 3(d–f) show three cross-sectional FIB cuts taken along the tapered gratings at the locations indicated by the red dotted lines in Fig. 3(b), the positions of which were determined by optical microscope inspection. Note that these three positions do not necessarily coincide with the top-down SEM measurements (black points). The redline overlays are the parameters determined by diffractometry at these three locations. The height of the structure matches well with the cross-sections, consistently determined to be 250 nm across the majority of the structures (Fig. S2(a)). Additionally, a 90° SWA was predicted (Fig. S2(b)) in agreement with the steep side-wall angles seen by top down SEM images of the structures.

### Height sensing

**Figure 4:**
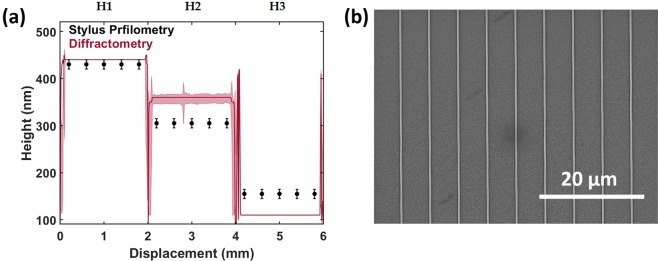
Variable height structures. (**a**) Height data obtained from diffractometry from samples H1, H2 and H3, stitched together at displacement 2 and 4 mm. The black points represent the average value of height obtained by stylus profilometry, 430 nm, 305 nm and 155 nm respectively. (**b**) Top-down SEM image of sample H1.

To test the robustness of this method against height variation, three linear gratings were fabricated with different etch depths, H1, H2 and H3. Copies of these structures were also designed with 10% of their lines missing to simulated fabricated defects, D1, D2 and D3 the results of which can be found in supporting information Fig. S4. Figure 4(a–b) show the diffractometry results and an SEM image for the sample H1. Each sample was scanned separately for inspection and the three separate results were stitched together at 2 and 4 mm in Fig. 4(a).

Comparing samples H1, H2 and H3, it is observed that when the inspection beam illuminates the sample, a constant height is measured with a small degree of uncertainty from the χ^2^ minimisation. Moving from samples H1, H2 and H3 the diffractometer measures heights of 440 nm, 360 nm and 110 nm respectively, using an EM library with a parametric resolution of ±10 nm. The black points and error bars show the height data as measured by stylus profilometry, 430 nm, 305 nm and 155 nm (±10 nm) respectively for H1, H2 and H3.

The obtained top-width (Fig. 4(b)) between all samples showed a relatively constant value, 140 or 160 nm, which corresponds well with SEM image analysis of the top (148 ± 9 nm). The combined SEM and stylus profilometry analysis for all samples estimates the SWA to be 78 ± 3°. This is in good agreement with diffractometry for H1 measuring a SWA of 80°. However, H2 and H3 deviate, with diffractometry measuring side-wall angles of 70° and 30° respectively (Fig. 4(c)). This inaccurate prediction of the SWA is believed to be the cause of why there is a 35 and 20 nm discrepancy between diffractometry and profilometry in samples H2 and H3 respectively. Most likely the simple trapezoidal modal is not describing the structures accurately enough, leading to inexact predictions of all the parameterised dimensions.

### Thermal nanoimprinted PMMA structures

A set of nine poly(methyl methacrylate) (PMMA) films was patterned by thermal nanoimprint lithography (NIL) using the structure H2 as the master. The imprinting temperature between the samples was varied from 120 °C to 200 °C in 10 °C steps, with the imprinting time kept constant at 3 minutes. This was done in order to inspect the produced structures above and below the glass transition temperature of the PMMA used (113 °C).

**Figure 5:**
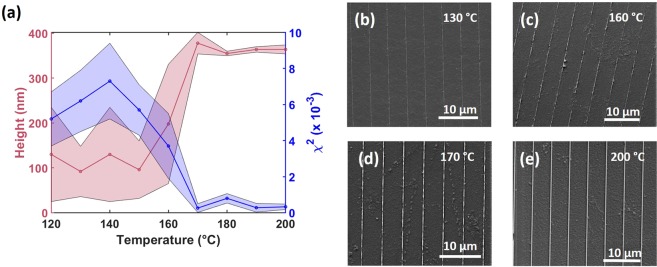
Thermally nanoimprinted structures. (**a**) Average height diffractometry data from the thermal nanoimprinted samples as a function of imprinting temperature (black) and the average minimum χ^2^ (red). (**b**–**e**) SEM image selection from samples imprinted at 130, 160, 170 and 200 °C, respectively.

Figure 5(a) shows the average height obtained via diffractometry when the thermal NIL samples were scanned underneath the inspection beam. The error bars plotted represent the standard deviation in measured heights for the entire scan. The minimum χ^2^ value from diffraction pattern look-up process are also plotted. Above 160 °C there is a clear jump in the height data from a broad uncertain prediction to a narrow average height range of 355–365 nm measured across the samples. These values are consistent with the height measured by diffractometry of the master structure H2 as seen in Fig. 4(a). Accompanying the jump in the height there is a notable drop in the χ^2^. As the structures begin to form under sufficient temperature by the thermal NIL process, they become uniform over a larger area and exhibit fewer defects, allowing the χ^2^ minimisation process to find an appropriate diffraction pattern in the library to match to the experimental data.

A selection of SEM images was taken across the sample set (shown in Fig. 5(b–e)) of samples imprinted at 130, 160, 170 and 200 °C respectively. These SEM images reveal that for an imprinting temperature of 160 °C and below, the PMMA structures are not well formed and contain many defects. Above this temperature the structures become better defined which coincides with the diffractometry results, and additionally, supports the fabrication rule of thermal NIL that the imprinting temperature should be around 50 °C above the glass transition temperature (T_g_ = 113 °C) for high fidelity.

## Discussion

In summary, we present a yet unexplored form of optical scatterometry for critical dimension metrology with nanoscale feature sizes; diffractometry. The technique works on the principle that the optical diffraction pattern can be used to determine the unit cell critical dimensions via inverse problem-solving whereby an imaged diffraction pattern is compared to a theoretically computed library and a χ^2^ minimisation process is used to predict the structural parameters in real-time. The method was tested on different silicon and PMMA structures fabricated by e-beam lithography and thermal nanoimprint lithography respectively. Additionally, thermal NIL samples fabricated under various imprinting temperatures were inspected, demonstrating the viability of diffractometry as quality control for nanoimprint lithography.

The tool was used to detect the variation of structural parameters of linear grating; height, width and side-wall angle. Diffractometry showed an extremely high sensitivity to structural and surface defects making it easy to identify high fidelity areas and those with a higher degree of defectivity. Averaged across all sample sets, the top-width and height measurements via diffractometry were accurate to 9.1% and 9.8% when compared to SEM images and stylus profilometry as references respectively. In most cases this equated to several tens of nanometres difference and is on the same order as the uncertainty of the cross-referencing method. Side-wall angles measurements however exhibited a much greater level of inaccuracy averaging to a 25% difference from those calculated via the combination of SEM and profilometry. This is believed to be due to the FDFD model not taking into account corner rounding and over-etching.

This technique differs from other forms of scatterometry in a variety of different ways. Principally this technique uses the diffraction efficiency as a function of diffraction order as opposed to wavelength or angle-of-incidence. As such a different computational technique had to be used to accurately predict all higher order orders of diffraction, namely, finite-difference frequency-domain (FDFD). FDFD has the advantage of being able to simulate realistic experimental conditions whereby a finite structure is computed with a Gaussian incident field. Future improvements can be made by moving to finite-element methods (FEM) which allow for accurate simulation of curved edges and slopped edges potentially improving the accuracy of the computational libraries.

Further to the presented work we have made initial attempts to integrate real-time diffractometry in-line to a UV-assisted roll-to-roll nanoimprint, laboratory scale tool. This has proved promising although technical issues of stability during the roll-to-roll NIL process have yet to be solved. We envisage diffractometry to become an important quality control inspection technique for high-volume microstructure production giving critical dimension accuracy down to ~10 nm.

## Materials and Methods

### Optical set-up

The optical set-up consisted of a fibre-coupled HeNe source collimated by an elliptical mirror, subsequently polarised to match the simulations, and then telescoped down to beam-size of 200 μm onto the sample stage, as shown in Fig. 1(a). The sample plane was positioned one focal length away from the aspherical objective (16 mm). The sample stage was motorised to control the scanning speed and to mimic a real-time inspection in a production line. All samples were measured at 1 mm/s scanning speed. The CCD camera is positioned in the Fourier plane of the sample stage in order to obtain a sharp diffraction pattern. The speed of measurement was limited to 100 ms, due to the internal 10 fps frame rate of the camera. The photodiode was positioned in a collimated section of the 0th reflected beam. Higher orders diffraction travelling off the optical axis were blocked off by a pin hole such that only the 0^th^ order was detected by the photodiode. The power measured from the photodiode was then used to normalise the data collected from the CCD such that the 0^th^ order diffraction was equal to unity (interfaced via LabVIEW). The analysed CCD images there for provides a one-dimensional array containing the diffraction intensity with respective to the 0^th^ order of each detected higher order of diffraction.

### Real-time metrology

The normalised observed diffraction efficiencies $({O}_{i}^{norm})$ from the CCD were fed into a simultaneously running MATLAB code which was used to compare the observed values to those in the EM library $\,(E{x}_{i})$. For each cell in the library a chi-squared value was calculated:

$${\chi }^{2}=\sum _{i}\frac{{({O}_{i}^{norm}-E{x}_{i})}^{2}}{E{x}_{i}}$$

where *i* denotes the order of diffraction.

The minimum value of in the generated chi-squared array was then located by converting the n-dimensional array into a 1D array, extracting the minimum value, and then reconverting it’s index back into the original parametric dimensions to identify the corresponding top width, height and SWA parameters from the EM-library. These are then given as the measured result of said parameter (solid coloured lines in Figs. 2(b,c), 3(b) and 4(a)). Additionally, the parameters for chi-squared cells within the relative experimental uncertainty of the minimum were generated to give an idea of experimental uncertainty in the measurement (filled circles in Figs. 2(b,c), 3(b) and 4(a)).

Due to the asymmetrical nature of the tapered structures, the diffraction patterns are degenerate for the first and second half of the grating lines. In order to measure the entire length of the structure two diffraction libraries had to be used, one for the first half, and another for the second; the two sets of results were later stitched together for presentation purposes at 35 mm displacement in Fig. 3(b).

### Stylus profilometry

To obtain a close approximation of the height for these thin films (~100 nm), a stylus profilometer (Alphastep D500, KLA Tencor inc.) was used for scanning at a near parallel angle to the grating lines, ~5° off axis. In this manner we could maximise the time spent by the scanning tip on the thin structures to get an accurate height reading. Attempts were made to use AFM for this cross reference, however, the height of the structures made accurate readings problematic.

### Si grating fabrication

The silicon structures were fabricated by electron beam lithography using a Vistec EBPG5000 system. A 550 nm thick PMMA 996k film was spin coated on a HMDS coated 4-inch Si wafer followed by a 6 minute pre-bake at 180 °C. A beam step size of 25 nm including proximity effect correction was used to achieve a quality of side wall roughness. Reactive ion etching was carried out to transfer the pattern into the Si substrate to the desired height.

### Thermal nanoimprint lithography

The thermal nanoimprint lithography (NIL) process was performed using a desktop equipment (CNI Tool from NIL Technology ApS), which allows imprinting of any stamp onto a substrate size of up to 10 cm in diameter. The substrates were 2×2 cm^2^ PMMA films with a thickness of 125 µm. Before the thermal NIL process, the silicon master stamp was treated with an anti-adhesion material, Optool™ DSX from Daikin Chemical Europe (Düsseldorf, Germany), in order to prevent possible adhesion. All samples were patterned using an imprinting time of 3 min and a pressure of 5 bar, while the imprinting temperature was varied from 120 °C to 200 °C in 10 °C steps; the separation temperature between stamp and substrate was fixed at 40 °C

## Supplementary information

Supplementary Information.
