# Supplementary material for: Real-time Optical Dimensional Metrology via Diffractometry for Nanofabrication
Source: Sci Rep. 2020 Mar 25;10:5371. doi: 10.1038/s41598-020-61975-3 (PMC7096422; doi:10.1038/s41598-020-61975-3)
Supplement: Supplementary file 1 — Supplementary Information. [file 41598_2020_61975_MOESM1_ESM.pdf]

Supplementary Information: Real-time Optical Dimensional Metrology via Diffractometry for Nanofabrication

Guy L. Whitworth[1,*], Achille Francone[1], Clivia M. Sotomayor-Torres[1,2] and N. Kehagias[1]

[1] Catalan Institute of Nanoscience and Nanotechnology (ICN2), CSIC and BIST, Campus UAB, 08193 Bellaterra, Barcelona, Spain
[2] Institucio Catalana de Recerca i Estudis Avancats (ICREA), 08010 Barcelona, Spain
*guy.whitworth@icfo.eu

1. Simulation Ranges of all Structures - SI

Table S1 Simulation range of metrological parameters for tested samples

Sample Code	Design	Height Range	Top Width Range	Side-Wall Angle Range
S	Step-Wise	80 nm – 150 nm	250 nm – 450 nm	40 ° – 90 °
T	Triangluar	200 nm – 280 nm	200 nm – 4800 nm	60 ° – 90 °
H	Height Variation	80 nm – 450 nm	100 nm – 200 nm	30 ° – 80 °
D	Defective	80 nm – 450 nm	100 nm – 200 nm	30 ° – 80 °
N	Nanoimprinted	80 nm – 450 nm	100 nm – 200 nm	30 ° – 80 °

2. Step-Wise Structures – SI

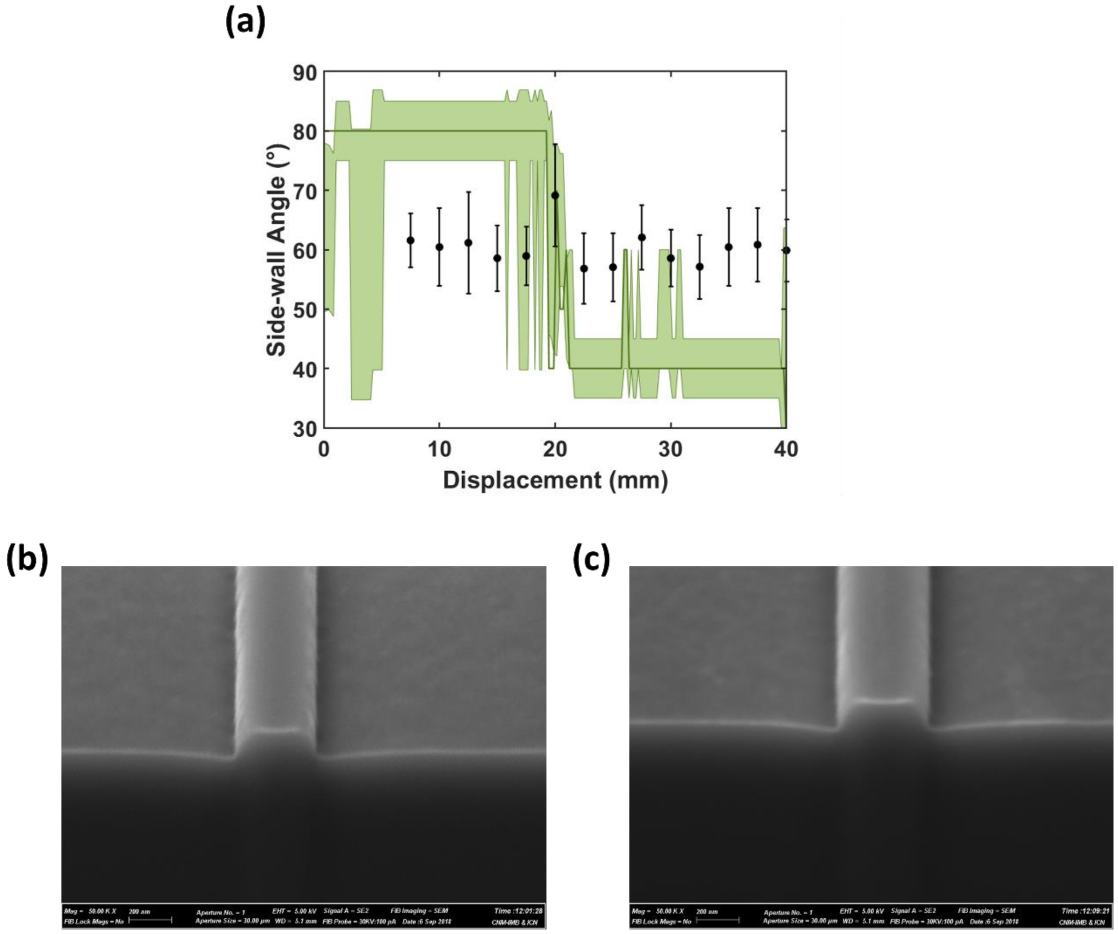

Fig. S1. (a) Diffractometry predictions of the SWA (green) of the S-series structures linked to the same measurements as in Fig2(b)-(c). Black points represent the SWA as calculated by combining stylus profilometry data of the height and the top and base widths of the structures obtained from SEM images. (b) and (c) show the original SEM images seen in Fid2(d)-(e) without the overlaid diffractometry predictions, where the over-etching can be seen more apparently.

3. Triangular Structures - SI

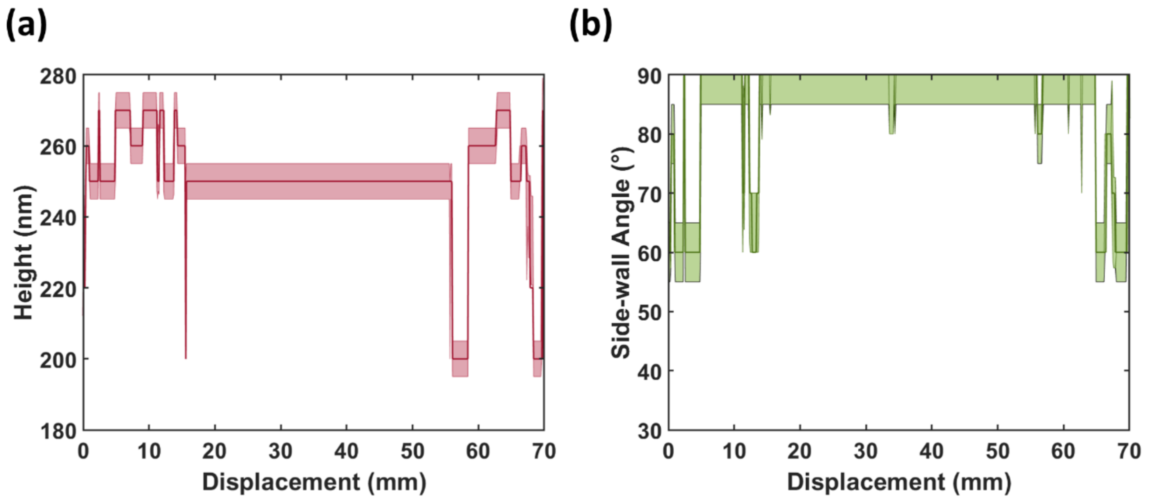

Fig. S2. Diffractometry results for **(a)** Height and **(b)** SWA data obtained from the trapezoidal structures. These are the counterpart results to the data shown in Fig3(b).

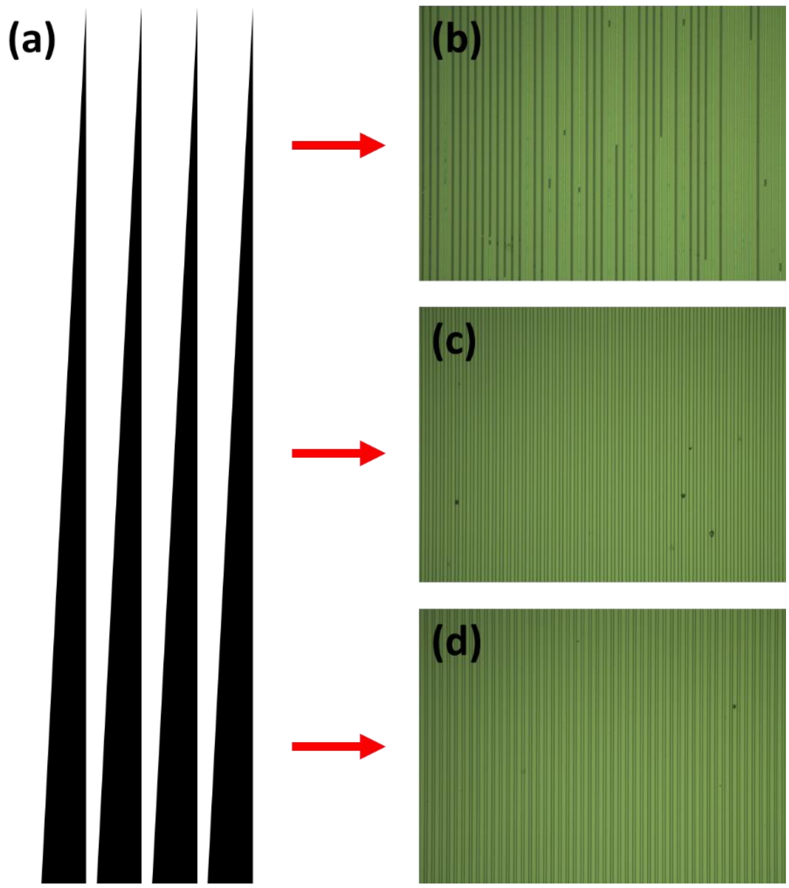

Fig. S3. (a) Reference diagram of trapezoidal structures. (b)-(d) Optical images of three areas along the trapezoids to highlight the surface defects in (a).

4. Height Varying Structures with added Defects - SI

Samples D1, D2 and D3 contain 10% missing lines as engineered defects (FigS4(e), (f) and (g)). When the inspection beam passes across the defective samples the diffraction signal varied rapidly, and as such, the metrological data as well changed in a likewise manner, especially for samples D1 and D3. For D2, the effect is less apparent due to degeneracy issues stated in the manuscript. The spread in the data for D2 however, is seen to be noisier and value of the width is pushed further away from the value obtained by SEM analysis. As such D1, D2 and D3 can be easily identified as defective when scanning for quality control.

Additional samples were also fabricated with 20% and 30% missing lines (data not presented), however since the signal was so rapidly degraded by only 10% lines missing no additional metrological data could be extracted from these. For future work more subtle defects should be introduced into the gratings as 10% missing lines was too much of a brutal change and doesn't push the boundaries of the technique's sensitivity.

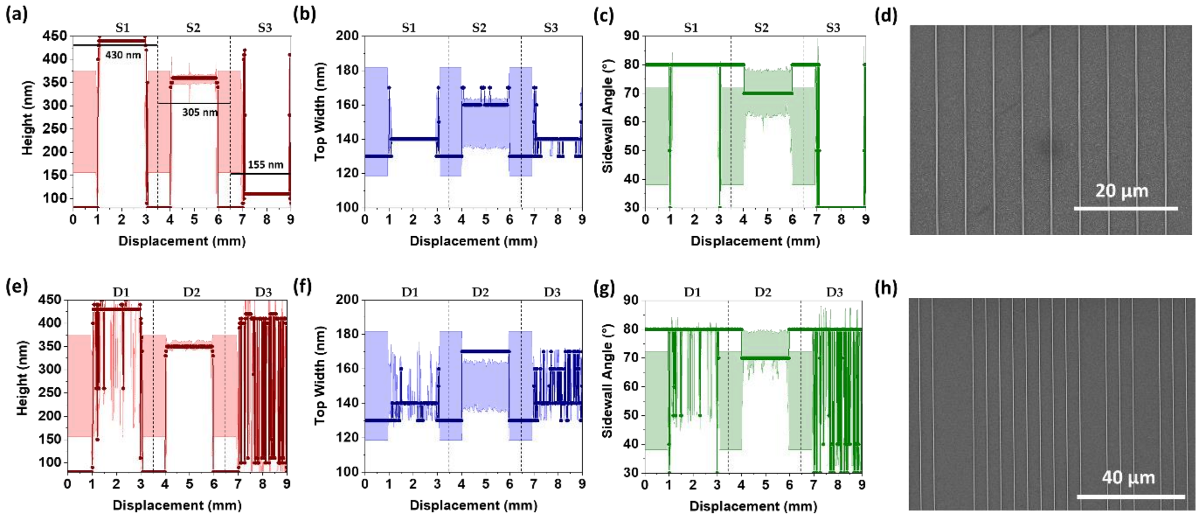

Fig. S4. (a)-(c) Show the Height, Top Width and Sidewall Angle respectively of the height varying samples S1, S2 and S3 with an examples SEM image (d). (e)-(g) Similarly shows the diffractometry results but from defective samples D1, D2 and D3 with an example SEM image (h).
